# Supplementary material for: COZOID: contact zone identifier for visual analysis of protein-protein interactions
Source: BMC Bioinformatics. 2018 Apr 6;19:125. doi: 10.1186/s12859-018-2113-6 (PMC5889581; doi:10.1186/s12859-018-2113-6)
Supplement: Supplementary file 2 — Software build. Executable binary file of the software tool. (ZIP 118,784 kb) [file 12859_2018_2113_MOESM2_ESM.zip › cozoid/java/docs/beansbinding-1.2.1-doc.zip!/org/jdesktop/el/impl/MethodExpressionImpl.html]

MethodExpressionImpl (Beans Binding)


---


|  |  |  |
| --- | --- | --- |
| **Overview** \| **Package** \| **Class** \| **Use** \| **Tree** \| **Deprecated** \| **Index** \| **Help** | |  |
| **PREV CLASS**   **NEXT CLASS** | **FRAMES**    **NO FRAMES**     **All Classes** |  |
| SUMMARY: NESTED \| FIELD \| CONSTR \| METHOD | DETAIL: FIELD \| CONSTR \| METHOD |  |


---


## org.jdesktop.el.impl Class MethodExpressionImpl

```
java.lang.Object
  org.jdesktop.el.Expression
      org.jdesktop.el.MethodExpression
          org.jdesktop.el.impl.MethodExpressionImpl
```

**All Implemented Interfaces:**: java.io.Externalizable, java.io.Serializable

---

``` public final class MethodExpressionImpl extends MethodExpression implements java.io.Externalizable ```

An `Expression` that refers to a method on an object.

`The ExpressionFactory.createMethodExpression(org.jdesktop.el.ELContext, java.lang.String, java.lang.Class , java.lang.Class []) method
can be used to parse an expression string and return a concrete instance
of MethodExpression that encapsulates the parsed expression.
The FunctionMapper is used at parse time, not evaluation time,
so one is not needed to evaluate an expression using this class.
However, the ELContext is needed at evaluation time.`

The `getMethodInfo(org.jdesktop.el.ELContext)` and `invoke(org.jdesktop.el.ELContext, java.lang.Object[])` methods will evaluate the
expression each time they are called. The `ELResolver` in the
`ELContext` is used to resolve the top-level variables and to
determine the behavior of the `.` and `[]`
operators. For any of the two methods, the `ELResolver.getValue(org.jdesktop.el.ELContext, java.lang.Object, java.lang.Object)`
method is used to resolve all properties up to but excluding the last
one. This provides the `base` object on which the method
appears. If the `base` object is null, a
`NullPointerException` must be thrown. At the last resolution,
the final `property` is then coerced to a `String`,
which provides the name of the method to be found. A method matching the
name and expected parameters provided at parse time is found and it is
either queried or invoked (depending on the method called on this
`MethodExpression`).

See the notes about comparison, serialization and immutability in
the `Expression` javadocs.

**See Also:**: `javax.el.ELResolver`, `javax.el.Expression`, `javax.el.ExpressionFactory`, `javax.el.MethodExpression`, Serialized Form

---

**Nested Class Summary**

**Nested classes/interfaces inherited from class org.jdesktop.el.Expression**

`Expression.ResolvedProperty, Expression.Result`


**Constructor Summary**

`MethodExpressionImpl()`

`MethodExpressionImpl(java.lang.String expr,
Node node,
FunctionMapper fnMapper,
VariableMapper varMapper,
java.lang.Class expectedType,
java.lang.Class[] paramTypes)`


| **Method Summary** | |
| --- | --- |
| `boolean` | `equals(java.lang.Object obj)`             Determines whether the specified object is equal to this `Expression`. |
| `java.lang.String` | `getExpressionString()`             Returns the original String used to create this `Expression`, unmodified. |
| `MethodInfo` | `getMethodInfo(ELContext context)`             Evaluates the expression relative to the provided context, and returns information about the actual referenced method. |
| `int` | `hashCode()`             Returns the hash code for this `Expression`. |
| `java.lang.Object` | `invoke(ELContext context, java.lang.Object[] params)`             Evaluates the expression relative to the provided context, invokes the method that was found using the supplied parameters, and returns the result of the method invocation. |
| `boolean` | `isLiteralText()`             Returns whether this expression was created from only literal text. |
| `void` | `readExternal(java.io.ObjectInput in)` |
| `void` | `writeExternal(java.io.ObjectOutput out)` |

**Methods inherited from class java.lang.Object**

`clone, finalize, getClass, notify, notifyAll, toString, wait, wait, wait`

**Constructor Detail**

### MethodExpressionImpl

```
public MethodExpressionImpl()
```

---


### MethodExpressionImpl

```
public MethodExpressionImpl(java.lang.String expr,
                            Node node,
                            FunctionMapper fnMapper,
                            VariableMapper varMapper,
                            java.lang.Class expectedType,
                            java.lang.Class[] paramTypes)
```

**Parameters:**: `expr` -: `node` -: `fnMapper` -: `expectedType` -: `paramTypes` -


**Method Detail**

### equals

```
public boolean equals(java.lang.Object obj)
```

:   Determines whether the specified object is equal to this
    `Expression`.

    The result is `true` if and only if the argument is not
    `null`, is an `Expression` object that is the
    of the same type (`ValueExpression` or
    `MethodExpression`), and has an identical parsed
    representation.

    Note that two expressions can be equal if their expression Strings are
    different. For example, `${fn1:foo()}` and
    `${fn2:foo()}` are equal if their corresponding
    `FunctionMapper`s mapped `fn1:foo` and
    `fn2:foo` to the same method.

    :   **Specified by:**: `equals` in class `Expression`
    :   **Parameters:**: `obj` - the `Object` to test for equality. **Returns:**: `true` if `obj` equals this `Expression`; `false` otherwise. **See Also:**: `Hashtable`, `Object.equals(java.lang.Object)`

---


### getExpressionString

```
public java.lang.String getExpressionString()
```

:   Returns the original String used to create this `Expression`,
    unmodified.

    This is used for debugging purposes but also for the purposes of
    comparison (e.g. to ensure the expression in a configuration file has not
    changed).

    This method does not provide sufficient information to re-create an
    expression. Two different expressions can have exactly the same
    expression string but different function mappings. Serialization should
    be used to save and restore the state of an `Expression`.

    :   **Specified by:**: `getExpressionString` in class `Expression`
    :   **Returns:**: The original expression String. **See Also:**: `javax.el.Expression#getExpressionString()`

---


### getMethodInfo

```
public MethodInfo getMethodInfo(ELContext context)
                         throws PropertyNotFoundException,
                                MethodNotFoundException,
                                ELException
```

:   Evaluates the expression relative to the provided context, and returns
    information about the actual referenced method.

    :   **Specified by:**: `getMethodInfo` in class `MethodExpression`
    :   **Parameters:**: `context` - The context of this evaluation **Returns:**: an instance of `MethodInfo` containing information about the method the expression evaluated to. **Throws:**: `java.lang.NullPointerException` - if context is `null` or the base object is `null` on the last resolution.: `PropertyNotFoundException` - if one of the property resolutions failed because a specified variable or property does not exist or is not readable.: `MethodNotFoundException` - if no suitable method can be found.: `ELException` - if an exception was thrown while performing property or variable resolution. The thrown exception must be included as the cause property of this exception, if available. **See Also:**: `javax.el.MethodExpression#getMethodInfo(javax.el.ELContext)`

---


### hashCode

```
public int hashCode()
```

:   Returns the hash code for this `Expression`.

    See the note in the `equals(java.lang.Object)` method on how two expressions can be
    equal if their expression Strings are different. Recall that if two
    objects are equal according to the `equals(Object)` method,
    then calling the `hashCode` method on each of the two
    objects must produce the same integer result. Implementations must take
    special note and implement `hashCode` correctly.

    :   **Specified by:**: `hashCode` in class `Expression`
    :   **Returns:**: The hash code for this `Expression`. **See Also:**: `equals(java.lang.Object)`, `Hashtable`, `Object.hashCode()`

---


### invoke

```
public java.lang.Object invoke(ELContext context,
                               java.lang.Object[] params)
                        throws PropertyNotFoundException,
                               MethodNotFoundException,
                               ELException
```

:   Evaluates the expression relative to the provided context, invokes the
    method that was found using the supplied parameters, and returns the
    result of the method invocation.

    :   **Specified by:**: `invoke` in class `MethodExpression`
    :   **Parameters:**: `context` - The context of this evaluation.: `params` - The parameters to pass to the method, or `null` if no parameters. **Returns:**: the result of the method invocation (`null` if the method has a `void` return type). **Throws:**: `java.lang.NullPointerException` - if context is `null` or the base object is `null` on the last resolution.: `PropertyNotFoundException` - if one of the property resolutions failed because a specified variable or property does not exist or is not readable.: `MethodNotFoundException` - if no suitable method can be found.: `ELException` - if an exception was thrown while performing property or variable resolution. The thrown exception must be included as the cause property of this exception, if available. If the exception thrown is an `InvocationTargetException`, extract its `cause` and pass it to the `ELException` constructor. **See Also:**: `javax.el.MethodExpression#invoke(javax.el.ELContext, java.lang.Object[])`

---


### readExternal

```
public void readExternal(java.io.ObjectInput in)
                  throws java.io.IOException,
                         java.lang.ClassNotFoundException
```

:   **Specified by:**: `readExternal` in interface `java.io.Externalizable`
:   **Throws:**: `java.io.IOException`: `java.lang.ClassNotFoundException`

---


### writeExternal

```
public void writeExternal(java.io.ObjectOutput out)
                   throws java.io.IOException
```

:   **Specified by:**: `writeExternal` in interface `java.io.Externalizable`
:   **Throws:**: `java.io.IOException`

---


### isLiteralText

```
public boolean isLiteralText()
```

:   **Description copied from class: `Expression`**
:   Returns whether this expression was created from only literal text.

    This method must return `true` if and only if the
    expression string this expression was created from contained no
    unescaped EL delimeters (`${...}` or
    `#{...}`).

    :   **Specified by:**: `isLiteralText` in class `Expression`
    :   **Returns:**: `true` if this expression was created from only literal text; `false` otherwise.


---


|  |  |  |
| --- | --- | --- |
| **Overview** \| **Package** \| **Class** \| **Use** \| **Tree** \| **Deprecated** \| **Index** \| **Help** | |  |
| **PREV CLASS**   **NEXT CLASS** | **FRAMES**    **NO FRAMES**     **All Classes** |  |
| SUMMARY: NESTED \| FIELD \| CONSTR \| METHOD | DETAIL: FIELD \| CONSTR \| METHOD |  |


---
